# Development and Validation of a Multidimensional Population-Based Healthy Aging Scale: Results From the China Health and Retirement Longitudinal Study

**DOI:** 10.3389/fmed.2022.853759

**Published:** 2022-02-14

**Authors:** Junling Gao, Jixiang Xu, Yingwei Chen, Yujie Wang, Bo Ye, Hua Fu

**Affiliations:** ^1^School of Public Health, Fudan University, Shanghai, China; ^2^Collaborative Innovation Cooperative Unit of National Clinical Research Center for Geriatric Diseases, Shanghai, China; ^3^Core Unit of Shanghai Clinical Research Center for Geriatric Diseases, Shanghai, China; ^4^Huadong Hospital, Fudan University, Shanghai, China

**Keywords:** healthy aging, validation, bi-factor model, psychometric evaluation, development

## Abstract

**Background:**

The World Health Organization proposed a multidimensional concept of healthy aging in 2015; there was limited evidence about how the concept was constructed and measured. The current study aims to develop a health aging scale (HAS) following the WHO framework and validate it using data from the China Health and Retirement Longitudinal Study (CHARLS).

**Methods:**

A total of 13,233 adults aged ≥ 45 years old from the CHARLS included in current study. Based on the WHO framework, 37 self-reported indicators were used to determine healthy aging. Exploratory factor analysis and second-order and bi-factor modeling, as well as psychometric coefficients, were used to examine the structure of healthy aging. To assess concurrent validity of the HAS, regression analyses were used to examine the associations of HAS and its subscales with sociodemographic characteristics, health conditions, healthcare utilization and life satisfaction in Wave 1. The predictive validity of HAS and subscales was assessed by their associations with mortality in Wave 2 follow-up using Cox regressions.

**Results:**

The general HAS and its five subscales were generated according to bi-factor modeling [CFI = 0.949; TLI = 0.942; SRMSR = 0.030; and RMSEA = 0.033 (95% CI, 0.032–0.034)] and psychometric coefficients (ω = 0.903; ωH = 0.692; ECV = 0.459). The general HAS presented solid evidence of concurrent validity with various sociodemographic characteristics, health conditions, healthcare utilization and life satisfaction; and predictive validity with mortality.

**Conclusions:**

The population-based multidimensional healthy aging scale and its subscales can be used to monitor the trajectories of general healthy aging and its subdomains to support the development of healthy aging policies and interventions.

## Introduction

The pace of population aging around the world is increasing dramatically (1). Between 2015 and 2050, the proportion of the world's population older than 60 years of age is expected to nearly double from 12 to 22% (1). China is one of the most rapidly aging countries; the newest census data indicated that there are more than 264 million people aged 60 years and older in 2020 living in China (2), accounting for 18.7% of the country's total population. By 2050, there will be almost 120 million aged 80 years or older living in China (1).

The world's rapidly aging population poses huge challenges to health and social care systems. To address these challenges, the World Health Organization (WHO) in 2015 proposed a public-health framework for healthy aging (3), which defined healthy aging as “the process of developing and maintaining the functional ability that enables well-being in older age.” Healthy aging is also one of the strategic objectives of the “global strategy and action plan on aging and health” adopted by the 69th World Health Assembly to measure, analyze, describe, and monitor healthy aging across the lifespan (4). Although many older adults may have one or more health conditions, which are well controlled and have little influence on their ability to function. Healthy aging pays more attention to an individual's functional ability across life-course. Functional ability is determined by an individual's intrinsic capacity, their environment, and the interaction of their intrinsic capacity and environment (5). Furthermore, a life-course approach to healthy aging has the potential to identify when and how to intervene at different life stages to maximize the chance of healthy aging for the population and for susceptible subgroups, and minimize variation by gender and socioeconomic group (6). As such, a healthy aging measure should broadly combine an individual's intrinsic capacity and functional ability and could sensitively capture the changes in a person's healthy aging level over life-course. The ideal study design for research taking a life course approach to healthy aging is a birth cohort (7), however most existing studies of aging have begun in middle or later age due to feasibility of implementation.

Although some epidemiological studies have used measures of healthy aging (8), these measures may fail to cover important domains of healthy aging (5, 8). Recently, researchers have attempted to develop healthy aging measures based on the WHO healthy aging framework. Sanchez-Niubo et al. used data from the Aging Trajectories of Health-Longitudinal Opportunities and Synergies (ATHLOS) project to develop the ATHLOS scale based on item response theory (IRT) modeling (9). Another newer healthy aging scale was developed using factor analysis methods employing data from six low- and middle-income Latin American countries (10). Theoretically speaking, healthy aging is a multidimensional concept that basically encompasses intrinsic capacity and functional ability (5). An empirical study using data from the English Longitudinal Study of Aging (ELSA) indicated that general factors (intrinsic capacity) and subdomain structure may contribute to a transformative paradigm for future research and clinical practice (11). However, no study has yet examined the multidimensionality of healthy aging. In addition, the existing healthy aging scales synthesized all items into one general index, which cannot provide more detail information on the specific dimensions of healthy aging. In the current study, we aimed to (1) examine the multidimensionality of healthy aging, and (2) develop and validate a healthy aging scale (HAS) and subscales following the WHO framework using data from the China Health and Retirement Longitudinal Study (CHARLS).

## Method

### Data Source

The CHARLS was a nationally representative longitudinal survey designed to examine health and economic adjustments due to rapid aging of the population in China. A more detailed description has been published elsewhere (12). In short, Wave 1 of CHARLS was conducted between June 2011 and March 2012 involving 17,708 respondents aged at least 45 years old who were followed up with every two years via a face-to-face computer-assisted personal interviews. Data of this study were drawn from Wave 1 of CHARLS, covering a total of 13,233 respondents after excluding those with missing data of HAS indicators. The ethical review committee at Peking University approved CHARLS.

### Indicators of Healthy Aging Scale

According to the WHO healthy aging framework (5), functional ability enables people to be and to do what they have reason to value, which refers to peoples' ability to meet (1) basic needs, (2) to learn, grow, and make decisions, (3) to be mobile, (4) to build and maintain relationships, and (5) to contribute to society. Intrinsic capacity comprises all the physical and mental capacities that a person can draw on, which includes: (1) locomotor capacity, (2) sensory capacity (such as vision and hearing), (3) vitality (energy and balance), (4) cognition, and (5) psychological capacity. Thirty-seven self-reported indicators related to intrinsic capacity and functional ability were selected to construct the HAS, which cover the following 6 dimensions of healthy aging: sensory capacity (4 indicators), psychological capacity (10 indicators), cognition capability (4 indicators), locomotion capacity (5 indicators), and activities of daily living (ADL, measuring ability to meet basic needs and to me mobile, 14 indicators). These indicators were measured using a five-point Likert scale, which was harmonized to be positive in the current study. Detailed information on the indicators' measurements can be found in Supplementary Table 1.

### Development of HAS

Firstly, considering the 37 indicators have different scales, we used min–max normalization to standardize the indicators to 0–1. Second, exploratory factor analysis (EFA) was used to identify the pattern of relationships between indicators and to decide the appropriate number of factors. Parsimax rotation, allowing for factor correlation and for minimum variable complexity, was employed to foster factor interpretability. Factor loadings of at least 0.20, in absolute value terms, were considered to establish a factor loading cutoff point (13).

**Figure 1:**
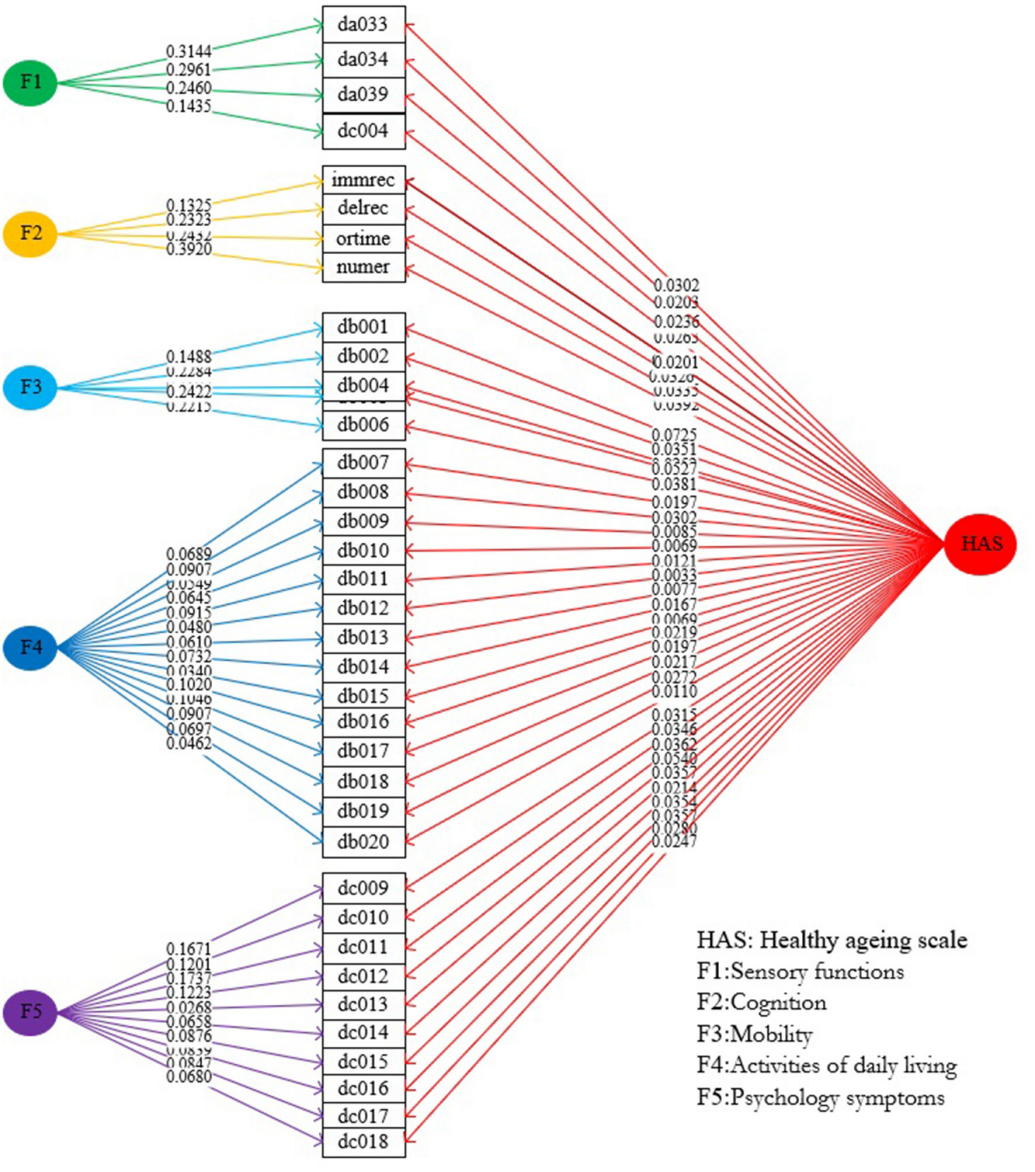
Weights of indicators on HAS and subscales.

**Figure 2:**
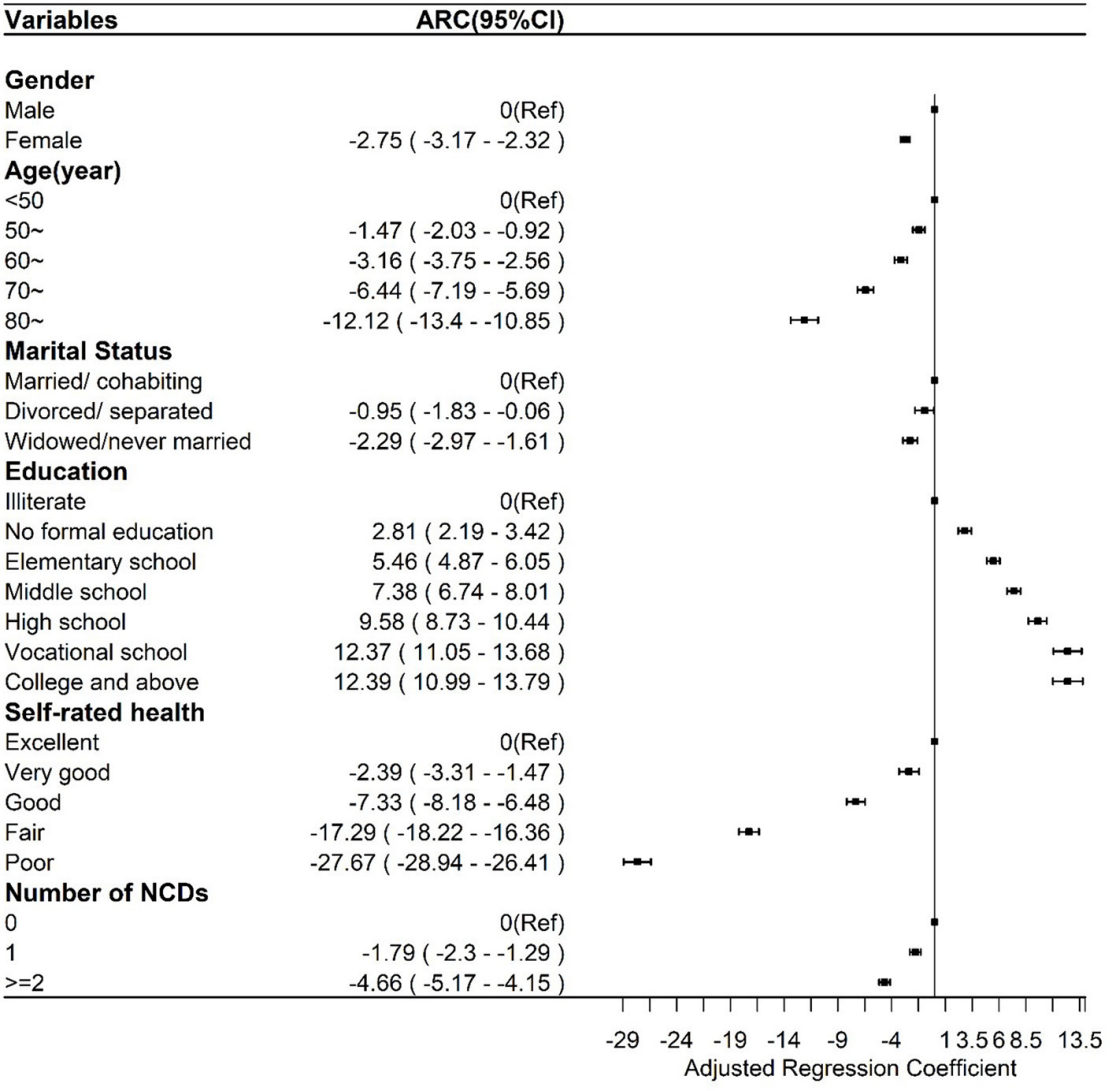
Multiple linear regression between the general healthy aging scale and sociodemographic and health factors.

Confirmatory second-order models and bi-factor models were considered, taking the multidimensionality of healthy aging and an overall target construct into account. Both the second-order model and the bi-factor model were set up by using the number and item structure of first-order and subdomain factors as suggested by the EFA. The second-order model was constituted by a second-order factor onto which the first-order factors of the EFA were loaded (Supplementary Material 2; Figure 1); while the bi-factor model was constituted by a general factor onto which all items were loaded and several orthogonal subdomain factors onto which items were loaded as suggested by the EFA (Supplementary Material 2; Figure 2). To establish the best-performing model, we firstly identified the best second-order model and bi-factor model, then compared the two best models; the one with better performance was subsequently used to build the HAS. Seventy percent of random samples of the total sample group was used for EFA and the remaining 30% of samples were used for CFA. Model fit was assessed using the root mean square error of approximation (RMSEA) (good fit < 0.08), standardized root mean square residual (SRMR) (good fit < 0.08), the comparative fit index (CFI) (good fit > 0.90), and the Tucker–Lewis index (TLI) (good fit > 0.90).

To further examine the multidimensionality of healthy aging, we calculated psychometric coefficients for the bi-factor model. We calculated omega (ω), omega hierarchical coefficient (ωH), coefficient omega hierarchical subscale (ωS), and explained common variance (ECV) because, in the bi-factor model, these indicators are assumed to be influenced by both the general factors and the specific factors (14). A high ω value indicates a highly reliable multidimensional composite, and a high ωH value (> 0.80) in the bi-factor structure indicates that the general factor is the dominant source of systematic variance, with subdomain factors having less influence. Meanwhile, the coefficient ωHS represents the proportion of reliable systematic variance of a subscale score after partitioning out general factor variability (15). Higher values of ECV indicate a strong general factor, thus allowing us to fit a unidimensional model to multidimensional data (16).

Finally, we calculated weights of indicators based on indicator loadings of the better of the two best models (17). Then, the HAS/its subscales were calculated by summing up products of the standardized indicators and their weights. HAS/all subscales were standardized using a range of zero to 100 points to make them easily comprehensible. Higher values indicate better healthy aging.

### Validation of HAS and Its Subscales

Validity was assessed in terms of concurrent validity and predictive validity. For concurrent validity, we firstly tested the associations of demographic characteristics, self-rated health, and numbers of non-communicable diseases (NCDs) with the HAS and its subscales using linear regression analyses. Secondly, we examined the associations of the HAS and its subscales with times of outpatient service (TOS), times of inpatient service (TIPS), and life satisfaction in Wave 1 using ordered logistic regression. Finally, the predictive validity of the HAS and its subscales was assessed by the association of the HAS in Wave 1 with mortality in Wave 2 using Cox regressions after controlling for demographic characteristics, self-rated health, and number of NCDs.

All statistical analysis was performed using R (R Foundation for Statistical Computing, Vienna, Austria) (18) and Stata version 13.0 (StataCorp LLC, College Station, TX, USA) (19).

## Results

### Sample Characteristics

After removing respondents with missing indicator data in the healthy aging scale, a total of 13,223 respondents from 28 provinces were included in the current study. As shown in Table 1, 47.52% (*n* = 6,284) of respondents were male, 44.28% were aged at least 60 years old, more than 80% were married/cohabiting, and 26.80% were illiterate. About half of respondents (49.41%) reported their health as “good”, and more than 60% of them were somewhat satisfied with their life. Only 32.55% of respondents (*n* = 4,151) had no NCDs, 37.80% had at least two NCDs, 9.33% used inpatient services in the past year, and 19.45% used outpatient services in the last month.

**Table 1:** Sample characteristics.

	***N***	**%**
**Sex**		
Male	6,284	47.52
Female	6,939	52.48
**Age (years)**		
<50	2,800	21.18
50~	4,568	34.55
60~	3,735	28.25
70~	1,719	13.00
80~	401	3.03
**Marital status**		
Married/cohabiting	11,114	84.05
Divorced/separated	686	5.19
Widowed/never married	1,423	10.76
**Education**		
Illiterate	3,543	26.80
No formal education	2,379	18.00
Elementary school	2,955	22.35
Middle school	2,743	20.75
High school	992	7.50
Vocational school	323	2.44
College and above	285	2.16
**Self-rated health**		
Excellent	791	5.98
Very good	2,266	17.14
Good	6,532	49.41
Fair	3,047	23.05
Poor	584	4.42
**Life satisfaction**		
Not at all satisfied	287	2.40
Not very satisfied	1,558	13.03
Somewhat satisfied	7,454	62.33
Very satisfied	2,437	20.38
Completely satisfied	222	1.86
**Times of inpatient care during the past year**		
0	11,988	90.67
1	963	7.28
≥2	271	2.05
**Times of outpatient service during the last month**		
0	10,637	80.55
1	1,388	10.51
≥2	1,180	8.94
**Numbers of NCD**		
0	4,151	32.55
1	3,781	29.65
≥2	4,820	37.80

### Development of HAS

#### Exploratory Factor Analysis

Extracting factors with eigenvalues of >1, the five-factor solution was deemed the best solution {CFI, 0.948; RMSR, 0.03; RMSEA, 0.049 [95% confidence interval (CI), 0.049–0.050]}. Four indicators of sensory function with loadings ranging from 0.45 to 0.74 were loaded on the first factor (eigenvalue = 1.57), which was labeled as sensory function. Four cognitive indicators with loadings ranging from 0.52 to 0.75 were loaded on the second factor (eigenvalue = 1.50), which was labeled as cognition. Five indicators of mobility with loadings ranging from 0.57 to 0.73 were loaded on the third factor (eigenvalue = 2.64), which was labeled as mobility. Fourteen indicators of ADL with loadings ranging from 0.39 to 0.83 were loaded on the fourth factor (eigenvalue = 5.41), which was labeled as ADL. Finally, 10 indicators of psychology symptoms with loadings ranging from 0.22 to 0.82 were loaded on the fifth factor (eigenvalue = 3.28), which was labeled as psychology capacity (Table 2).

**Table 2:** Standardized loadings of exploratory factor analysis.

**Items/Indicators**	**Factor 1**	**Factor 2**	**Factor 3**	**Factor 4**	**Factor 5**
da033	0.74				
da034	0.68				
da039	0.60				
dc004	0.45				
numer		0.52			
oritime		0.54			
delrec_01		0.58			
immrecall		0.75			
db001			0.57		
db002			0.66		
db004			0.58		
db005			0.73		
db006			0.65		
db007				0.39	
db008			0.32	0.39	
db009				0.49	
db010				0.8	
db011				0.83	
db012				0.81	
db013				0.72	
db014			0.26	0.54	
db015				0.47	
db016				0.69	
db017				0.67	
db018				0.60	
db019				0.40	
db020				0.43	
dc009					0.73
dc010					0.60
dc011					0.82
dc012					0.59
dc013					0.22
dc014					0.52
dc015					0.4
dc016					0.5
dc017					0.6
dc018					0.59

#### Confirmatory Factor Analysis

Both the second-order factor model and the bi-factor model exhibited good fit, but the bi-factor model exhibited higher CFI and TLI values and lower SRMR and RMSEA values [bi-factor model: CFI, 0.949; TLI, 0.942; SRMR, 0.030; RMSEA, 0.033 (95% CI, 0.032–0.034) vs. second-order model: CFI, 0.962; TLI, 0.926; SRMR, 0.041; RMSEA, 0.038 (95% CI: 0.037–0.039)]. In addition, the adjusted chi-squared test for model comparison supported the superiority of the bi-factor model as its value was significant relative to that of the second-order model (χ^2^ = 1,007.8; df = 30; *P* < 0.001). Therefore, the bi-factor model was employed in subsequent analyses.

#### Psychometric Coefficients

The general HAS showed good reliability (ω = 0.903) and based on the bi-factor model, ωH (ωH = 0.692) indicated both the general HAS and the subdomain factors need to be reported. A comparison of ωH and ω (0.84/0.96 = 0.88) showed that most of the reliable variance in total scores could be attributed to the general factor. Meanwhile, 21.1% of the reliable variance in total scores could be attributed to the multidimensionality caused by subdomain factors, and only 9.7% was estimated to be random errors. The omega hierarchy of the five subscale coefficients was in the order of 0.420, 0.336, 0.263, 0.609, and 0.450, indicating that some common variance remained after accounting for the general HAS. ECV was 0.459, also indicating that both the general HAS and the subdomain factors need to be reported.

#### Weight Assignment and Calculation of HAS and Its Subscales

Based on the best bi-factor model, we calculated the effects of all indicator weights (ranging from 0.0033 to 0.0724) on the general HAS and the corresponding indicator weights on subscales (Figure 1). The four indicator weights of the subscale of sensory function ranged from 0.1435 to 0.3144, the four indicator weights of the subscale of cognition ranged from 0.1325 to 0.3920, the five indicator weights of the subscales of mobility ranged from 0.1488 to 0.2422, the 14 indicator weights of the subscale of ADL ranged from 0.0340 to 0.1046, and the 10 indicator weights of the subscale of psychology ranged from 0.0268 to 0.1737. Finally, scores for the general HAS and its five subscales were calculated using the standardized indicators and their weights. Overall scores were as follows: 71.07 points (95% CI, 70.81–71.33 points) for the general HAS; and 31.72 points (95% CI, 31.43–32.02 points), 57.64 points (95% CI, 57.19–58.10 points), 81.03 points (95% CI, 80.65–81.42 points), 95.48 points (95% CI, 95.28–95.67 points), and 70.71 points (95% CI, 70.32–71.10 points) for the subscales of sensory function, cognition, mobility, ADL, and psychology, respectively. Detailed information about the distributions of scores of the general HAS and its subscales among demographic characteristics can be found in Supplementary Material 3.

### Validity of HAS

As shown in Figure 2, the general HAS scores of women [Adjusted Regression Coefficients (ARC), −2.75; 95% CI, −3.17 to −2.32] were lower than those of men, and the general HAS scores among those who were divorced/separated and widowed/never married were lower than the scores of those who were married/cohabiting. The general HAS score significantly increased with an increase in the education level, while it decreased with older age, presence of more NCDs, and poorer self-rated health. The relationship patterns of all subscale scores to the above factors resembled the patterns of the general HAS (Supplementary Material 4; Supplementary Figure 4a), except for no association between marital status and sensory function subscale. The scores of the cognition subscale of women (ARC = −2.21; 95% CI, −3.04 to −1.38) were lower than those of men, and scores of the cognition subscale among those widowed/never married (ARC, −3.05; 95% CI, −4.38 to −1.72) were lower than scores among those married/cohabiting. The cognition subscale scores were significantly higher with higher education level but lower with older age and worse self-rated health, while there was no association with the number of NCDs (Supplementary Material 4; Supplementary Figure 4b). The mobility subscale scores of women (ARC, −4.45; 95% CI, −5.14 to −3.76) were lower than those of men, and the mobility subscale scores among those widowed/never married (ARC, −1.24; 95% CI, −2.36 to −0.13) were lower than those of those married/cohabiting. The mobility subscale scores were significantly higher with higher education level but lower with older age and presence of more NCDs and worse self-rated health (Supplementary Material 4; Supplementary Figure 4c). Neither sex nor marital status was associated with ADL subscale scores, which were significantly higher with higher education level but lower with older age and presence of more NCDs and worse self-rated health (Supplementary Material 4; Supplementary Figure 4d). Except for the finding that psychology subscale scores among those aged 70 years or older were higher than the scores among those aged younger than 70 years, the relationship patterns of all other factors were consistent with the patterns of the general HAS (Supplementary Material 4; Supplementary Figure 4e).

**Figure 3:**
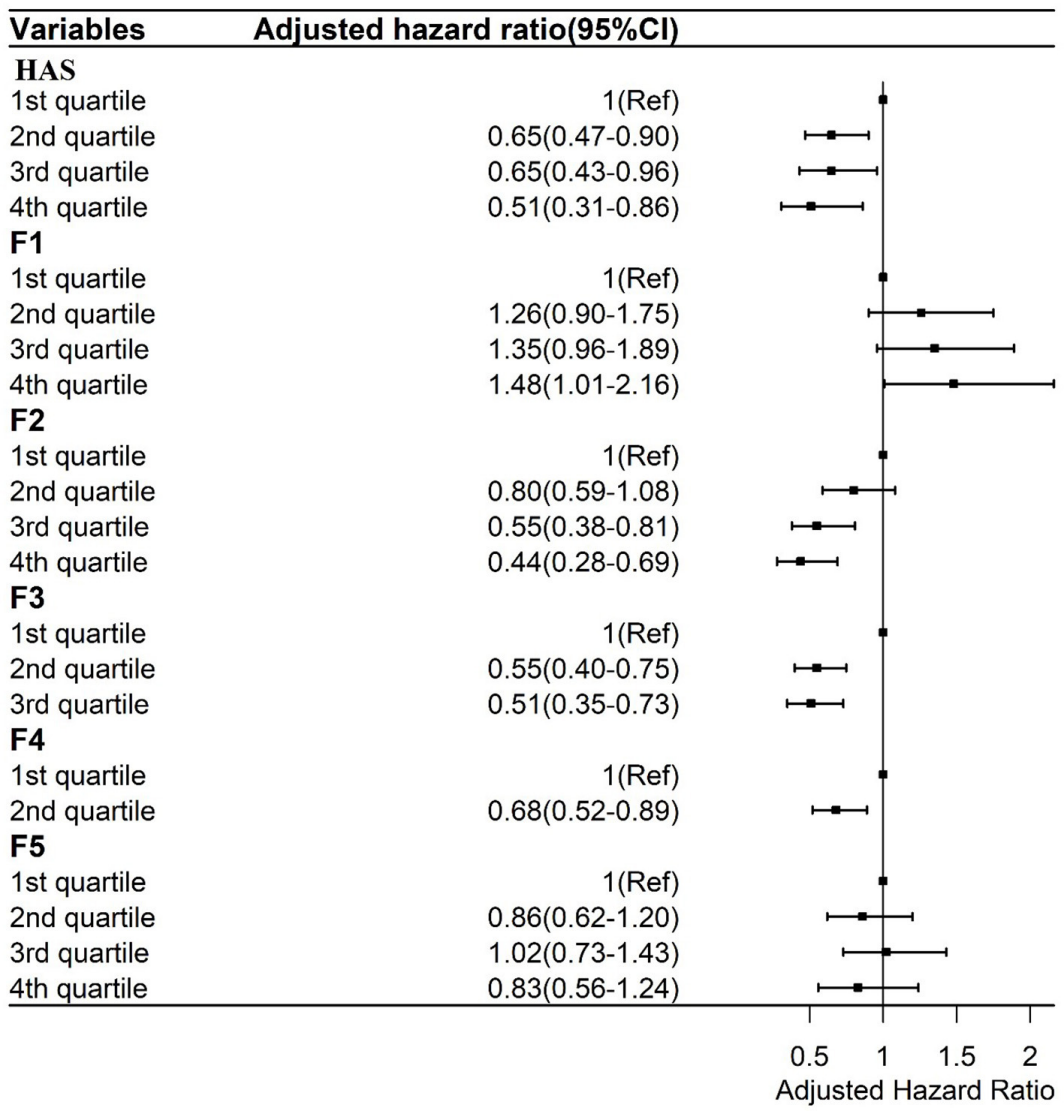
Cox regressions between the general HAS and its subscales and mortality adjusted by sociodemographic and health factors. F1: Sensory function scale; F2: Cognition scale; F3: Mobility Scale; F4: ADL scale; F5: Psychology scale.

The general HAS and its subscales were also divided into score quartiles to analyze their associations with mortality, TOS, TIPS, and life satisfaction. As shown in Figure 3, after controlling for demographic characteristics, self-rated health, and the number of NCDs, compared to those in the lowest score quartile of general HAS scores, those in the second [Adjusted Hazard Ratio (AHR), 0.65; 95% CI, 0.47–0.90], third (AHR, 0.65; 95% CI, 0.43–0.96), and fourth (AHR, 0.51; 95% CI, 0.31–0.86) score quartiles had a lower AHR of mortality.

Those in the highest score quartile of the sensory function subscale (**F1**) had a higher AHR value (1.48; 95% CI, 1.01–2.16) than those in the lowest score quartile. Those in the third (AHR, 0.55; 95% CI, 0.38–0.81) and fourth (AHR, 0.44; 95% CI, 0.28–0.69) score quartiles of the cognition subscale had lower AHR values of mortality than those in the lowest score quartile of the cognition subscale (**F2**). Those in the second (AHR, 0.55; 95% CI, 0.40–0.75) and third (AHR, 0.51; 95% CI, 0.35–0.73) score quartiles of the mobility subscale had lower AHR values of mortality than those in the lowest score quartile (**F3**). Finally, those in the second (AHR, 0.68; 95% CI, 0.52–0.89) score quartile of the ADL subscale had lower AHR values of mortality than those in the first score quartile (**F4**). There was no association between the psychology subscale and AHR values of mortality (**F5**).

**Figure 4:**
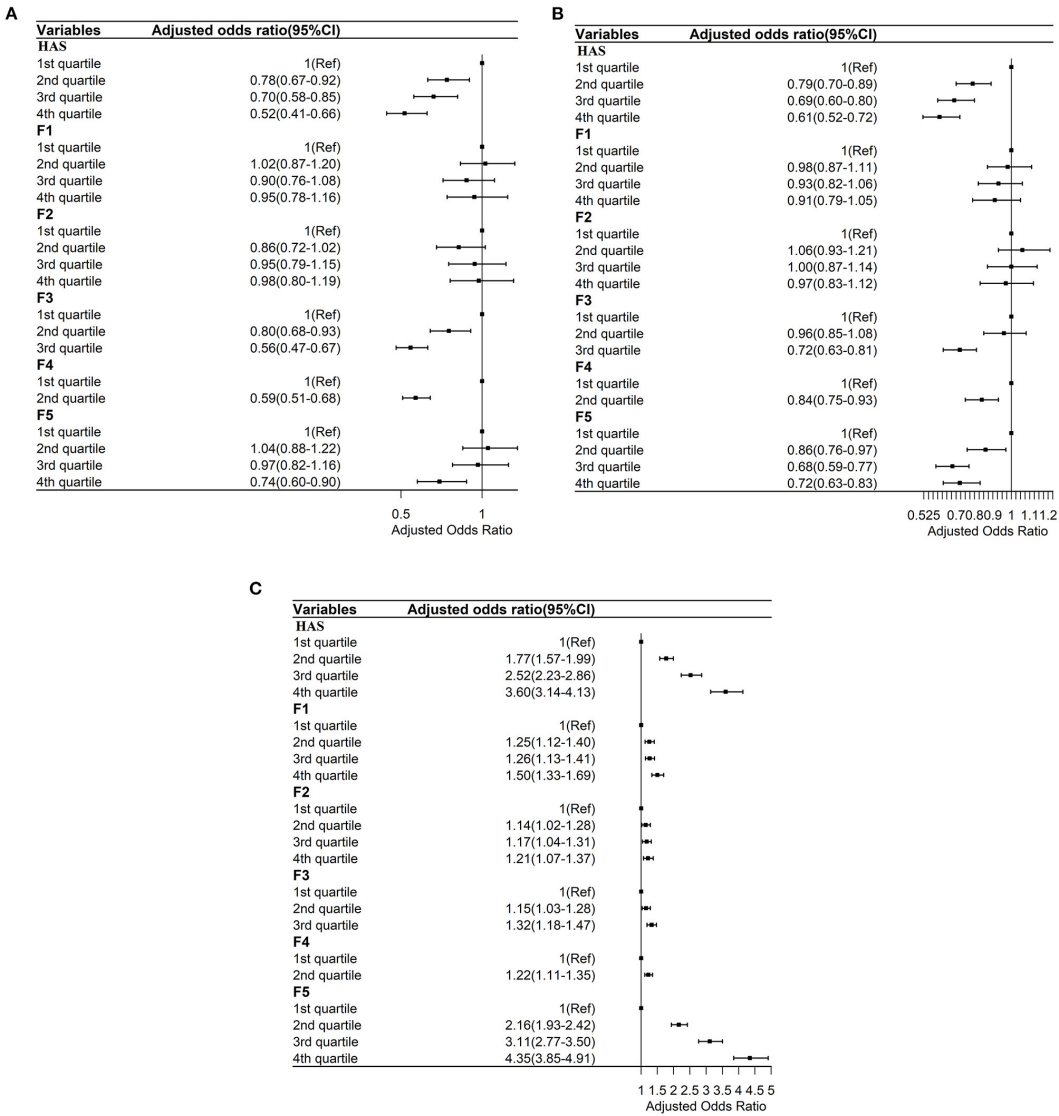
**(A)** Ordered logistic regressions between the general HAS and its subscales with times of inpatient service adjusted by sociodemographic and health factors. F1: Sensory function scale; F2: Cognition scale; F3: Mobility Scale; F4: ADL scale; F5: Psychology scale. **(B)** Ordered logistic regressions between the general HAS and its subscales with times of outpatient service adjusted by sociodemographic and health factors. F1: Sensory function scale; F2: Cognition scale; F3: Mobility Scale; F4: ADL scale; F5: Psychology scale. **(C)** Ordered logistic regressions between the general HAS and its subscales with life satisfaction adjusted by sociodemographic and health factors. F1: Sensory function scale; F2: Cognition scale; F3: Mobility Scale; F4: ADL scale; F5: Psychology scale.

As shown in Figure 4A, after controlling for demographic characteristics, self-rated health, and number of NCDs, compared to those in the lowest score quartile of the general HAS, those in the second [Adjusted Odds Ratio (AOR), 0.78; 95% CI, 0.67–0.92], third (AOR, 0.70; 95% CI, 0.58–0.85), and fourth (AOR, 0.52; 95% CI, 0.41–0.66) score quartiles had lower adjusted odds ratios of TIPS. Also, those in the second (AOR, 0.80; 95% CI, 0.68–0.93) and third (AOR, 0.56; 95% CI, 0.47–0.67) score quartiles of the mobility subscale (**F3**) had lower AOR values of TIPS than those in the lowest score quartile (**F4**). Those in the second (AOR, 0.59; 95% CI, 0.51–0.68) score quartile of the ADL subscale had lower AOR values of TIPS than those in the first score quartile of the ADL subscale. Finally, those in the fourth (AOR, 0.74; 95% CI, 0.60–0.90) score quartile of the psychology subscale had lower AOR values of TIPS than those in the first score quartile (**F5**).

As shown in Figure 4B, after controlling for demographic characteristics, self-rated health, and number of NCDs, compared to those in the lowest score quartile of the general HAS, those in the second (AOR, 0.79; 95% CI, 0.70–0.89), third (AOR, 0.69; 95% CI, 0.60–0.80), and fourth (AOR, 0.61; 95% CI, 0.52–0.72) score quartiles had lower adjusted odds ratios of TOS. Those in the third (AOR, 0.72; 95% CI, 0.63–0.81) score quartile of the mobility subscale (**F3**) had lower AOR values of TOS than those in the lowest score quartile of the mobility subscale. Those in the second (AOR, 0.84; 95% CI, 0.75–0.93) score quartile of the ADL subscale (**F4**) had lower AOR values of TOS than those in the first score quartile of the ADL subscale. Those in the third (AOR, 0.68; 95% CI, 0.59–0.77) and fourth (AOR, 0.72; 95% CI, 0.63–0.83) score quartiles of the psychology subscale had lower AOR values of TOS than those in the first score quartile of the psychology subscale (**F5**).

As shown in Figure 4C, after controlling for demographic characteristics, self-rated health, and number of NCDs, higher scores on the general HAS and all subscales were positively associated with higher AOR of a higher level of life satisfaction.

## Discussion

According to the WHO's model, healthy aging is a multidimensional concept that includes both intrinsic capacity and functional ability (5), which was also confirmed by a previous study (10). Previous studies have demonstrated that intrinsic capacity is multidimensional, including psychological, sensory, cognitive, vitality, and locomotor components (11, 20). In this study, we used data from a large national longitudinal study to firstly conceptualize healthy aging as a general factor and five subdomain factors, as identified by the EFA. Then, the multidimensionality of healthy aging was confirmed by CFA using a confirmatory bi-factor model. Based on the bi-factor model, we also calculated psychometric coefficients (15), which indicated that total score variance is caused by the general HAS and its subdomain factors. Taken together, we conclude that both the general HAS and the subdomain factors need to be reported. Although different indicators were included to develop the HAS, our findings are consistent with previous studies that concluded healthy aging is a multidimensional concept (10, 20).

To the best of our knowledge, there exist three studies that have previously created healthy aging scales using data from existing large longitudinal studies. Sanchez-Niubo et al. used item response theory to develop a unidimensional healthy aging scale including a total of 41 items, using data harmonized and integrated from 16 international cohorts (9). Daskalopoulou et al. demonstrated the multidimensionality of healthy aging but created a unidimensional healthy aging index with 26 items using bi-factor analysis methods, using data from six low- and middle-income Latin American countries (10). Another study (21) scored six health indicators from the CHARLS data as zero (healthiest), one, or two (unhealthiest) point(s) and summed them to construct the Chinese Healthy Aging Index (total score range, 0–12 points). Weighting indicators are a critically important issue to develop a multidimensional index (22). Weights of indicators should reflect their relative importance in their contributions to a multidimensional index. Factor analysis assigns the weights based on the factor loadings on the extracted components (23). The largest factor loadings are allocated to the indicators with the largest variation across the dataset, and vice versa. Factor analysis is suitable for comparison, the analysis of large datasets, and no required a priori assumptions or information on the weights of indicators (24). Thus, we assigned the weights of indicators based on a bi-factor model and calculated the scores of the general HAS and five subscales (sensory, cognition, mobility, ADL, and psychology) according to their item weights. Creating the general HAS and specific domain subscales not only can help in monitoring the general status of healthy aging but also its specific domains, which can provide more detailed information for developing healthy aging policies.

One key consideration of healthy aging is to reduce inequity (25), so a reliable healthy aging scale should be sensitive to change over time (5) and variations in sociodemographic characteristics of older adults. Although they employed different methods, previous studies have indicated that substantial variation exists in healthy aging across sociodemographic characteristics (8, 21, 26, 27) including sex, age, education, marital status, and income/wealth. Research has also found that sociodemographic characteristics are associated with healthy aging trajectories (28–30). Consistent with previous findings, our study also found that the scores of the general HAS and its subscales have good sensitivity to capture differences between sociodemographic groups. Self-rated health was negatively associated with scores of the general HAS and all subscales, consistent with previous studies (10, 31). Multiple morbidities represent one of the challenges affecting older adults (32) and have negative impacts on functional ability (33, 34) and successful aging (35). Similarly, we also found that the number of NCDs was negatively associated with scores of the general HAS and sensory function, mobility, ADL, and psychology subscales.

In order to further validate the general HAS and its subscales, we first examined their predictive ability for mortality as in previous works (36–38). We similarly found that the general HAS had good predictive ability for mortality. We also found three subscales—cognition, mobility and ADL—to have some predictive ability of mortality, which is consistent with previous studies (39, 40). Studies showed that some domains of healthy aging, such as physical functional impairment (41, 42), mobility limitations and cognitive deficits (43), and disability (44), are risk factors for utilization of healthcare, but no study has examined the association of healthy aging and healthcare utilization. In the current study, we also found that the general HAS and some subscales (mobility, ADL, and psychology) were negatively associated with healthcare utilization. Finally, subjective wellbeing (including life satisfaction, hedonic wellbeing, and eudemonic wellbeing) is important at older ages (45), and this is a key component of healthy aging (46). Research has shown that sensory function (47), cognitive ability (48), disabilities (49, 50), executive function (51), mobility (52), and psychology are associated with life satisfaction among older adults. Besides confirming the previous findings, we also found that the general HAS was progressively and positively associated with life satisfaction. Interestingly, we found that older adults with the greatest sensory function had higher AHR values (1.48; 95% CI, 1.01–2.16) than those with the poorest sensory function. The reason for this may be that the mortality patterns between these two groups of elders are different, which needs to be explored further in the future.

Our study had several strengths. First, we created a multidimensional healthy aging scale and subscales based on the WHO conceptual framework (1, 5), which can provide more detailed information to support healthy aging policies. Second, a large-scale, nationally representative study sample was used, which may facilitate the possible generalization of our findings. Third, our multidimensional healthy aging scale was rigorously validated by examining variations according to sociodemographic characteristics; its associations with self-rated health and the number of NCDs; and its prediction of mortality, healthcare utilization, and life satisfaction.

However, our study also has several limitations. First, to “build and maintain relationships” and “contribute to society” are important domains of healthy aging (5), which were not included in our multidimensional healthy aging scale due to lack of data. Second, a reliable healthy aging scale should be sensitive to changes in trajectories over time, which was beyond the scope of this study and further analyses are highly recommended.

## Conclusions

In conclusion, this study confirmed the multidimensionality of healthy aging and developed and validated a population-based multidimensional healthy aging scale and associated subscales. Our findings found that the general healthy aging scale and its subscales were variational according to sociodemographic characteristics; they were also associated with self-rated health and the number of NCDs; and they have reliable predictive ability for mortality, healthcare utilization, and life satisfaction. These findings imply the general healthy aging scale and its subscales might be used to monitor the trajectories of general healthy aging and its subdomains, facilitating the development of healthy aging policies and interventions. Our findings also found that sensory capacity and cognition was lower than mobility, ADL, and psychology among Chinese older adults. Policies and services should pay greater attention to promotion of sensory function and cognition among Chinese older adults. In addition, it is warranted to monitor trajectory of healthy aging and to explore its determinants in the future.

## Data Availability Statement

The raw data supporting the conclusions of this article will be made available by the authors, without undue reservation.

## Ethics Statement

The studies involving human participants were reviewed and approved by the Ethical Review Committee at Peking University approved CHARLS. The patients/participants provided their written informed consent to participate in this study.

## Author Contributions

JX, YC, and YW were responsible for the data cleaning. JX, BY, and JG analyzed and interpreted data. JG and HF wrote the draft. All authors revised and approve the final manuscript.

## Funding

This work was supported by the National Key R&D Program of China (Grant Nos. 2018YFC2002000 & 2018YFC2002001) and National Natural Science Foundation of China (Grant No. 82173634).

## Conflict of Interest

The authors declare that the research was conducted in the absence of any commercial or financial relationships that could be construed as a potential conflict of interest.

## Publisher's Note

All claims expressed in this article are solely those of the authors and do not necessarily represent those of their affiliated organizations, or those of the publisher, the editors and the reviewers. Any product that may be evaluated in this article, or claim that may be made by its manufacturer, is not guaranteed or endorsed by the publisher.
