# Supplementary material for: Development and Validation of a Multidimensional Population-Based Healthy Aging Scale: Results From the China Health and Retirement Longitudinal Study
Source: Front Med (Lausanne). 2022 Feb 14;9:853759. doi: 10.3389/fmed.2022.853759 (PMC8882972; doi:10.3389/fmed.2022.853759)
Supplement: Supplementary file 2 [file Data_Sheet_2.pdf]

Methods of developing healthy ageing scale

Given the relative importance degree of the indicator to the healthy ageing scale, so we synthetized healthy ageing indicators into one scale based on indicators' weights. The steps are as following:

Step 1 Data standardization: because the 37 indicators have different scales, we used min-max normalization to standardize the indicators into 0-1, and remove units.

$$x'_{ji} = \frac{x_{ji} - x_{min}}{x_{max} - x_{min}} \text{ (Formula 1)}$$

x_{ji}: The raw value of ith indicator of jth dimensionality

x'_{ji}: The standardized value of ith indicator jth dimensionality

x_{max}: The maximal raw value of ith indicator of all respondents
x_{min}: The minimal raw value of ith indicator of all respondents

Step 2 Weight assignment of indicators and calculating HAI: the weights of indicators were calculated according to the standardized loading coefficients of indicators of the better best model.

If the second-order model was the better best model (Figure 1), we use Formula 2 and Formula 3 to calculate weights of factors and indicators respectively based their standardized loadings, than use Formula 4 to calculate HAI.

$$\omega_{fj} = \frac{\beta_{fj}}{\sum_{i=1}^{i} \beta_{fj}} \text{ (Formula 2)}$$

$$\omega_{ji} = \frac{\lambda_{ji}}{\sum_{i=1}^{i} \lambda_{ji}} \text{ (Formula 3)}$$

$$\text{HAI} = 100 * \sum_{j=1}^{j} \omega_{fj} \sum_{i=1}^{i} \omega_{ji}\, x'_{ji} \text{ (Formula 4)}$$

ω_{fj}: the weight of jth factor on HAI
β_{fj}: The loading of jth factor on HAI
ω_{ji}: the weight of ith indictor on jth factor (dimensionality)
λ_{ji}: the loading of ith indictor on jth factor (dimensionality)

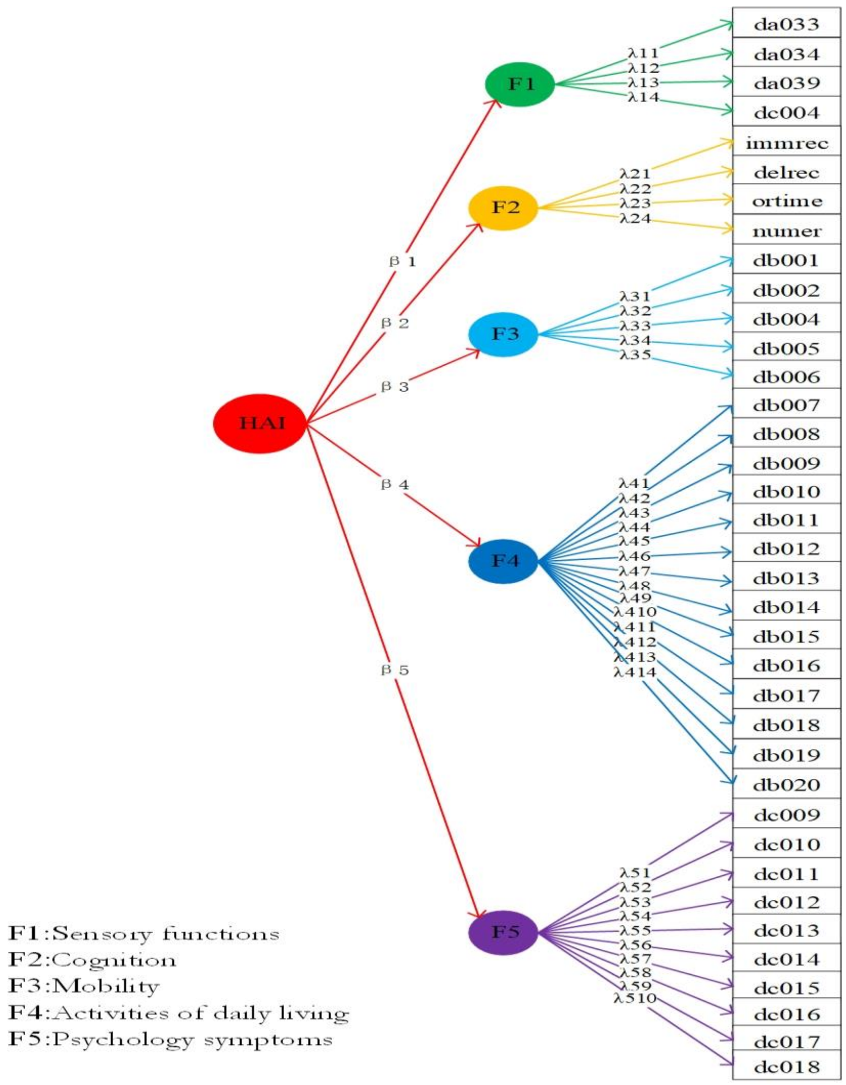

Figure 1 the standardized loading of the best second-order model

If the bifactor model was the better best model (Figure 2), we use Formula 5 and Formula 6 to calculate weights of factors and indicators respectively based their standardized loadings, than use Formula 7 to calculate HAI and Formula 8 to calculate subscale (dimensionality).

$$\omega_{gi} = \frac{\lambda_{gi}}{\sum_{i=1}^{i} \lambda_{gi}} \quad \text{(Formula 5)}$$

$$\omega_{ji} = \frac{\lambda_{ji}}{\sum_{i=1}^{i} \lambda_{ji}} \quad \text{(Formula 6)}$$

$$\text{HAI} = 100 * \sum_{i=1}^{i} \omega_{gi}\, x_i' \quad \text{(Formula 7)}$$

$$\text{Scale}_j = 100 * \sum_{i=1}^{i} \omega_{ji}\, x_{ji}' \quad \text{(Formula 8)}$$

ω_{gi}: the weight of ith indicator on HAI
λ_{gi}: The loading of ith indicator on HAI
ω_{ji}: the weight of ith indictor on jth factor (dimensionality)
λ_{ji}: the loading of ith indictor on jth factor (dimensionality)

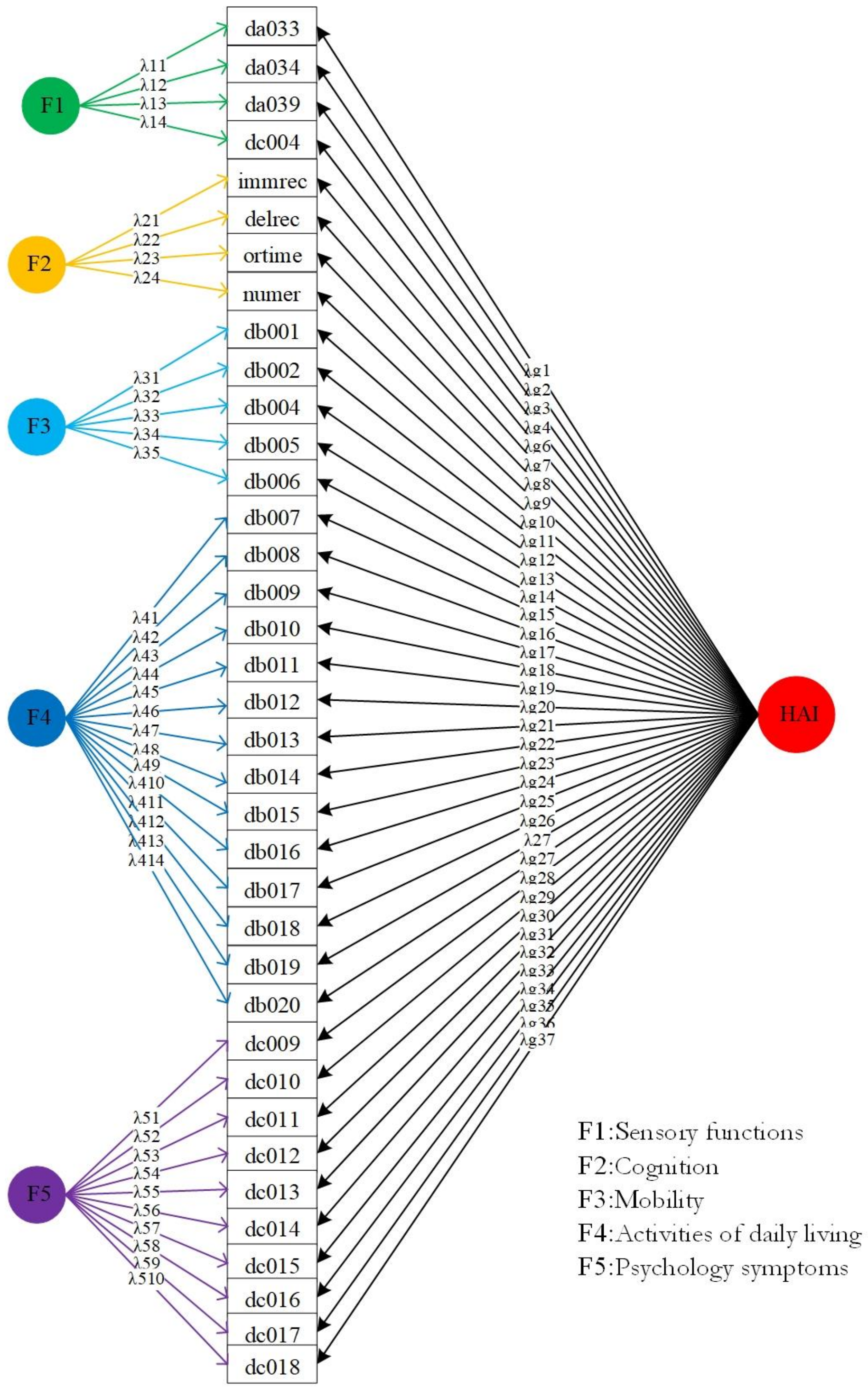

Figure 2 the standardized loading of the best bifactor model
