# Supplementary material for: Development and Validation of a Multidimensional Population-Based Healthy Aging Scale: Results From the China Health and Retirement Longitudinal Study
Source: Front Med (Lausanne). 2022 Feb 14;9:853759. doi: 10.3389/fmed.2022.853759 (PMC8882972; doi:10.3389/fmed.2022.853759)
Supplement: Supplementary file 3 [file Data_Sheet_3.pdf]

Supplementary S3 distributions of the general HAS and subscales among demographic characteristics

	HAS		F1		F2		F3		F4		F5	
	Mean(95%CI)	P value	Mean(95%CI)	P value	Mean(95%CI)	P value	Mean(95%CI)	P value	Mean(95%CI)	P value	Mean(95%CI)	P value
Total	71.07 (70.81; 71.33)		31.72 (31.43; 32.02)		57.64(57.19; 58.10)		81.03 (80.65; 81.42)		95.48 (95.28; 95.67)		70.71 (70.32; 71.10)	
**Gender**												
Male	74.18 (73.83; 74.53)	<.001	33.41 (32.97; 33.84)	<.001	62.99 (62.38; 63.60)	<.001	84.55 (84.03; 85.08)	<.001	96.18 (95.91; 96.45)	<.001	74.50 (73.98; 75.03)	<.001
Female	68.26 (67.90; 68.63)		30.20 (29.80; 30.61)		52.80 (52.16; 53.44)		77.85 (77.29; 78.40)		94.84 (94.57; 95.12)		67.27 (66.71; 67.84)	
**Age(year)**												
<50	76.73 (76.27; 77.19)	<.001	37.95 (37.28; 38.62)	<.001	65.66 (64.78; 66.55)	<.001	89.02 (88.41; 89.64)	<.001	98.23 (97.97; 98.48)	<.001	73.52 (72.72; 74.32)	<.001
50~	73.05 (72.64; 73.46)		32.36 (31.87; 32.84)		60.24 (59.51; 60.97)		84.05 (83.92; 85.08)		96.86 (96.59; 97.12)		71.44 (70.77; 72.11)	
60~	69.19 (68.71; 69.67)		29.42 (28.88; 29.96)		56.69 (55.85; 57.54)		78.73 (78.00; 79.46)		95.02 (94.66; 95.38)		68.76 (68.01; 69.51)	
70~	64.09 (63.30; 64.87)		26.68 (25.88; 27.48)		45.31 (44.05; 46.58)		69.10 (67.83; 70.36)		91.20 (90.44; 91.95)		69.58 (68.48; 70.69)	
80~	56.52 (54.73; 58.30)		24.14 (22.46; 25.83)		33.76 (31.27; 36.26)		58.32 (55.41; 61.22)		83.24 (81.10; 85.39)		65.79 (63.39; 68.19)	
**Marital Status**												
Married/ cohabiting	72.13 (71.85; 72.40)	<.001	32.20 (31.87; 32.52)	<.001	59.40 (58.92; 59.89)	<.001	82.19 (81.78; 82.60)	<.001	95.88 (95.67; 96.08)	<.001	71.79 (71.36; 72.21)	<.001
Divorced/ separated	70.69 (69.66; 71.73)		32.65 (31.27; 34.02)		56.19 (54.31; 58.07)		83.19 (81.71; 84.67)		96.76 (96.16; 97.37)		66.88 (65.13; 68.64)	
Widowed/never married	63.02 (62.18; 63.86)		27.59 (26.69; 28.85)		44.61 (43.19; 46.02)		70.98 (69.63; 72.32)		91.74 (90.95; 92.53)		64.16 (62.88; 65.43)	
**Education**												
Illiterate	63.17 (62.64; 63.69)	<.001	27.40 (26.84; 27.96)	<.001	37.50 (36.71; 38.29)	<.001	72.94 (72.10; 73.79)	<.001	92.23 (91.75; 92.71)	<.001	65.06 (64.25; 65.88)	<.001
No formal education	68.15 (67.56; 68.74)		28.85 (28.19; 29.52)		54.23 (53.25; 55.20)		78.71 (77.81; 79.62)		94.92 (94.45; 95.39)		66.66 (65.69; 67.62)	
Elementary school	72.46 (71.98; 72.94)		31.53 (30.94; 32.12)		62.31 (61.49; 63.14)		82.56 (81.81; 83.31)		96.50 (96.15; 96.86)		71.63 (70.84; 72.42)	
Middle school	76.79 (76.32; 77.26)		35.74 (35.09; 36.40)		70.63 (69.84; 71.41)		86.66 (85.93; 87.39)		97.49 (97.16; 97.82)		75.16 (74.38; 75.93)	
High school	79.82 (79.08; 80.56)		38.01 (36.91; 39.12)		75.41 (74.19; 76.63)		89.87 (88.81; 90.92)		98.18 (97.69; 98.66)		78.33 (77.10; 79.56)	
Vocational school	80.68 (79.48; 81.87)		38.56 (36.58; 40.53)		77.27 (75.30; 79.23)		88.60 (86.50; 90.69)		97.83 (96.99; 98.67)		81.63 (79.78; 83.48)	
College and above	83.10 (82.02; 84.19)		43.14 (40.98; 45.31)		79.26 (77.18; 81.34)		91.93 (90.27; 93.59)		98.63 (97.92; 99.34)		83.35 (81.46; 85.24)	
**Self-rated health**												
Excellent	83.03(82.35; 83.71)	<.001	49.51 (48.12; 50.90)	<.001	64.56 (62.79; 66.32)	<.001	93.90 (92.99; 94.82)	<.001	99.12 (98.85; 99.40)	<.001	84.91 (83.79; 86.03)	<.001
Very good	79.98 (79.53; 80.42)		41.24 (40.56; 41.92)		63.14 (62.08; 64.20)		91.67 (91.04; 92.29)		98.46 (98.21; 98.72)		81.47 (80.74; 82.19)	
Good	73.34 (73.05; 73.64)		30.80 (30.40; 31.17)		59.33 (58.71; 59.96)		84.63 (84.18; 85.08)		97.39 (97.21; 97.57)		73.24 (72.75; 73.74)	
Fair	60.73 (60.20; 61.25)		24.21 (23.65; 24.76)		50.65 (49.69; 51.61)		67.57 (66.68; 68.45)		91.33 (90.80; 91.86)		58.49 (57.66; 59.33)	
Poor	48.94 (47.59; 50.29)		20.23 (18.93; 21.54)		44.51 (42.35; 46.66)		52.47 (50.12; 54.83)		79.34 (77.36; 81.36)		45.06 (43.03; 47.09)	
**Numbers of NCD**												

0	77.08 (76.70; 77.46)		36.69 (36.15; 37.23)		60.58 (59.79; 61.38)		89.59 (89.07; 90.12)		98.00 (97.78; 98.23)		77.60 (76.98; 78.21)	
1	72.22 (71.77; 72.67)	<.001	32.40 (31.85; 32.95)	<.001	57.40 (56.56; 58.25)	<.001	82.73 (82.06; 83.41)	<.001	96.40 (96.08; 96.71)	<.001	71.94 (71.23; 72.64)	<.001
2	65.32 (64.88; 65.77)		27.37 (26.91; 27.83)		55.85 (57.39; 58.31)		72.69 (72.00; 73.39)		92.73 (92.32; 93.13)		64.03 (63.35; 64.71)	
