# Supplementary material for: Development and Validation of a Multidimensional Population-Based Healthy Aging Scale: Results From the China Health and Retirement Longitudinal Study
Source: Front Med (Lausanne). 2022 Feb 14;9:853759. doi: 10.3389/fmed.2022.853759 (PMC8882972; doi:10.3389/fmed.2022.853759)
Supplement: Supplementary file 4 [file Data_Sheet_4.pdf]

Variables	ARC(95%CI)
**Gender**	
Male	0(Ref)
Female	-1.6 (-2.18 - -1.02)
**Age(year)**	
<50	0(Ref)
50~	-4.26 (-5.01 - -3.5)
60~	-5.5 (-6.32 - -4.69)
70~	-7.64 (-8.67 - -6.61)
80~	-9.57 (-11.32 - -7.83)
**Marital Status**	
Married/ cohabiting	0(Ref)
Divorced/ separated	0.57 (-0.64 - 1.79)
Widowed/never married	0.23 (-0.7 - 1.16)
**Education**	
Illiterate	0(Ref)
No formal education	0.23 (-0.61 - 1.07)
Elementary school	1.56 (0.75 - 2.37)
Middle school	3.5 (2.64 - 4.36)
High school	5.27 (4.1 - 6.45)
Vocational school	7.95 (6.15 - 9.75)
College and above	9.53 (7.61 - 11.44)
**Self-rated health**	
Excellent	0(Ref)
Very good	-7.89 (-9.16 - -6.63)
Good	-17.06 (-18.23 - -15.89)
Fair	-21.79 (-23.06 - -20.52)
Poor	-24.86 (-26.59 - -23.12)
**Number of NCDs**	
0	0(Ref)
1	-0.95 (-1.64 - -0.26)
>=2	-2.81 (-3.51 - -2.12)

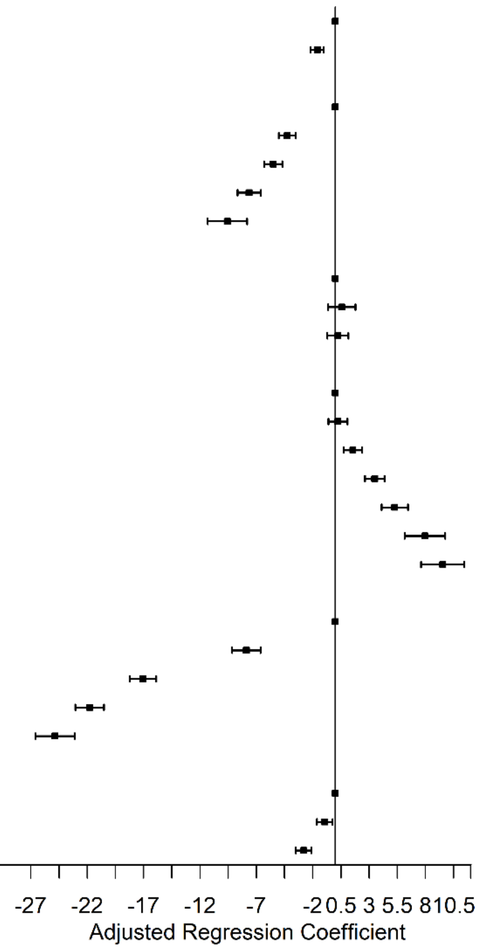

Figure S4a. Multiple linear regression between the sensory function scale and sociodemographic and health factors.

Variables	ARC(95%CI)
**Gender**	
Male	0(Ref)
Female	-2.21 (-3.04 - -1.38)
**Age(year)**	
<50	0(Ref)
50~	-1.17 (-2.25 - -0.08)
60~	-1.76 (-2.93 - -0.58)
70~	-8.47(-9.95--7.00)
80~	-15.36 (-17.86 - -12.86)
**Marital Status**	
Married/ cohabiting	0(Ref)
Divorced/ separated	-1.64 (-3.38 - 0.10)
Widowed/never married	-3.05 (-4.38 - -1.72)
**Education**	
Illiterate	0(Ref)
No formal education	14.24 (13.04 - 15.45)
Elementary school	21.43 (20.26 - 22.59)
Middle school	28.11 (26.87 - 29.34)
High school	32.81 (31.13 - 34.5)
Vocational school	35.77 (33.18 - 38.36)
College and above	36.43 (33.69 - 39.18)
**Self-rated health**	
Excellent	0(Ref)
Very good	-0.88 (-2.69 - 0.94)
Good	-2.92 (-4.6 - -1.25)
Fair	-7.58 (-9.4 - -5.76)
Poor	-11.8 (-14.29 - -9.32)
**Number of NCDs**	
0	0(Ref)
1	-0.62 (-1.61 - 0.37)
>=2	0.27 (-0.73 - 1.27)

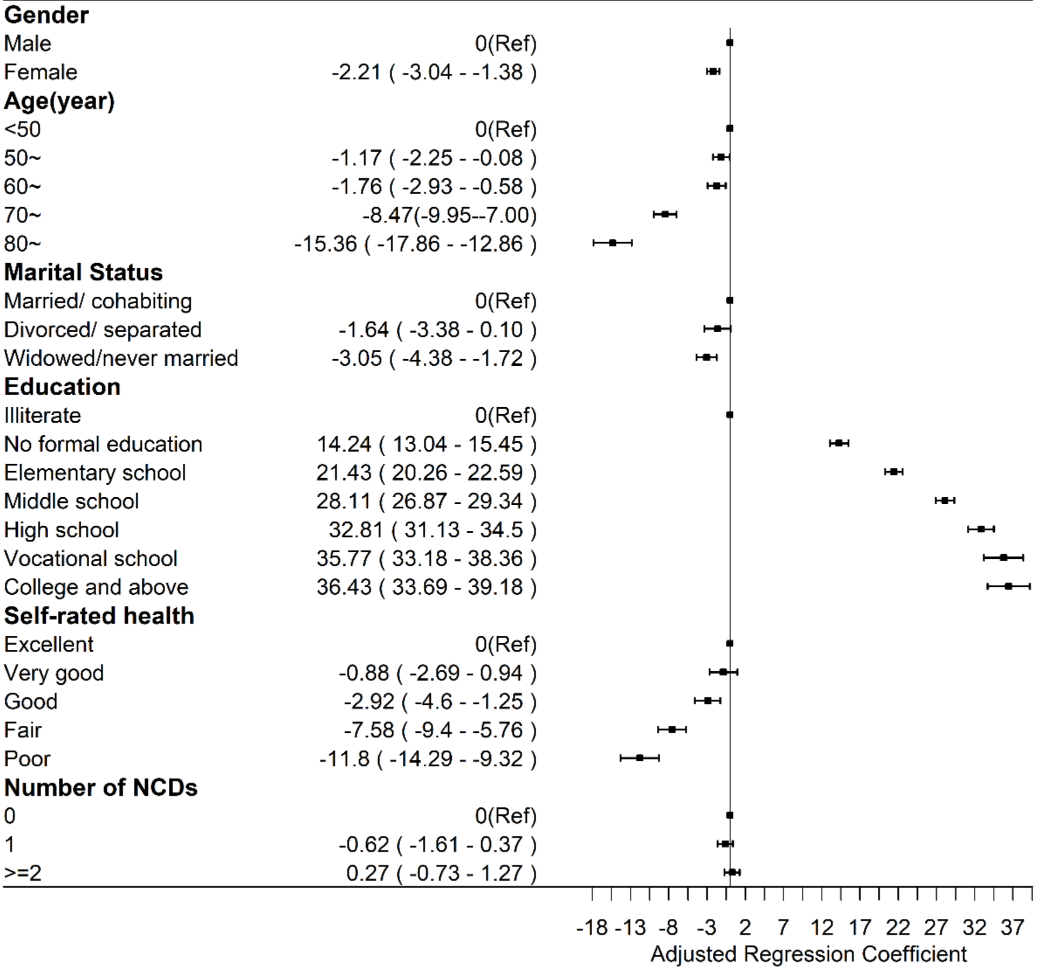

Figure S4b. Multiple linear regression between the cognition scale and sociodemographic and health factors.

Variables	ARC(95%CI)
**Gender**	
Male	0(Ref)
Female	-4.45 (-5.14 - -3.76)
**Age(year)**	
<50	0(Ref)
50~	-2.65 (-3.55 - -1.74)
60~	-6.26 (-7.24 - -5.28)
70~	-14.45 (-15.68 - -13.22)
80~	-23.67 (-25.76 - -21.58)
**Marital Status**	
Married/ cohabiting	0(Ref)
Divorced/ separated	0.93 (-0.52 - 2.38)
Widowed/never married	-1.24 (-2.36 - -0.13)
**Education**	
Illiterate	0(Ref)
No formal education	2.15 (1.14 - 3.15)
Elementary school	3.67 (2.7 - 4.64)
Middle school	3.83 (2.8 - 4.86)
High school	5.67 (4.27 - 7.08)
Vocational school	8.85 (6.69 - 11.01)
College and above	8.39 (6.1 - 10.68)
**Self-rated health**	
Excellent	0(Ref)
Very good	-1.26 (-2.77 - 0.25)
Good	-5.91 (-7.31 - -4.51)
Fair	-19.34 (-20.86 - -17.82)
Poor	-32.57 (-34.65 - -30.5)
**Number of NCDs**	
0	0(Ref)
1	-3.23 (-4.06 - -2.41)
>=2	-8.04 (-8.88 - -7.21)

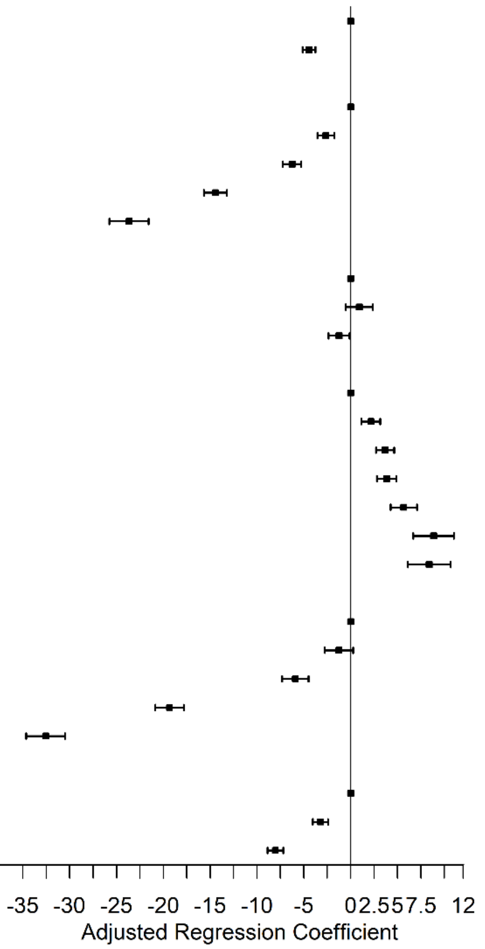

Figure S4c. Multiple linear regression between the mobility scale and sociodemographic and health factors.

Variables	ARC(95%CI)
**Gender**	
Male	0(Ref)
Female	-0.38 (-0.76 - 0.00)
**Age(year)**	
<50	0(Ref)
50~	-0.40 (-0.90 - 0.10)
60~	-1.57 (-2.11 - -1.04)
70~	-4.65 (-5.33 - -3.97)
80~	-11.76 (-12.91 - -10.62)
**Marital Status**	
Married/ cohabiting	0(Ref)
Divorced/ separated	0.64 (-0.16 - 1.44)
Widowed/never married	-0.36 (-0.97 - 0.25)
**Education**	
Illiterate	0(Ref)
No formal education	1.47 (0.92 - 2.03)
Elementary school	2.19 (1.66 - 2.72)
Middle school	1.93 (1.36 - 2.50)
High school	2.09 (1.32 - 2.87)
Vocational school	3.17 (1.99 - 4.36)
College and above	3.03 (1.77 - 4.28)
**Self-rated health**	
Excellent	0(Ref)
Very good	-0.31 (-1.15 - 0.52)
Good	-0.82 (-1.58 - -0.05)
Fair	-5.83 (-6.66 - -4.99)
Poor	-17.02 (-18.15 - -15.88)
**Number of NCDs**	
0	0(Ref)
1	-0.44 (-0.90 - 0.01)
>=2	-2.06 (-2.52 - -1.60)

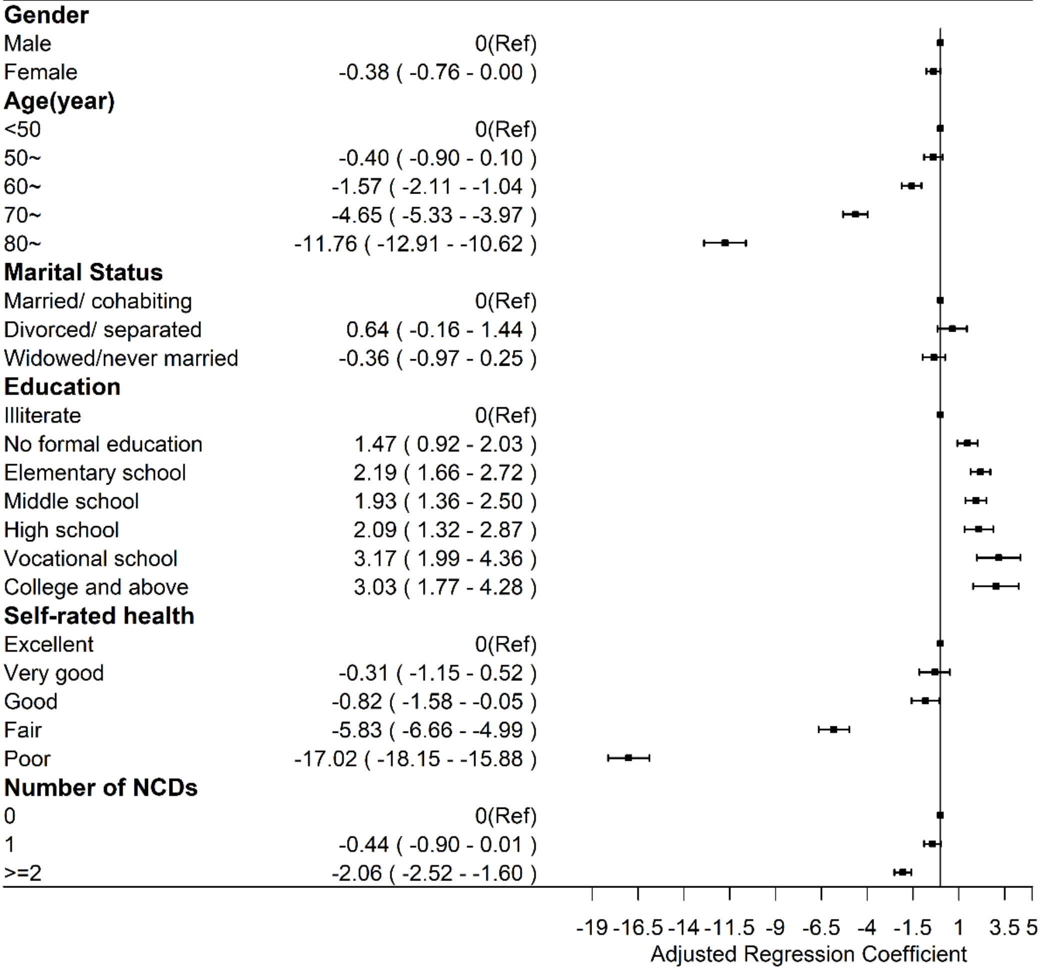

Figure S4d. Multiple linear regression between the ADL scale and sociodemographic and health factors.

Variables	ARC(95%CI)
**Gender**	
Male	0(Ref)
Female	-3.85 (-4.61 - -3.09)
**Age(year)**	
<50	0(Ref)
50~	0.20 (-0.79 - 1.20)
60~	0.04 (-1.03 - 1.12)
70~	2.67 (1.32 - 4.02)
80~	0.64 (-1.65 - 2.93)
**Marital Status**	
Married/ cohabiting	0(Ref)
Divorced/ separated	-3.73 (-5.32 - -2.13)
Widowed/never married	-4.22 (-5.44 - -3.00)
**Education**	
Illiterate	0(Ref)
No formal education	0.08 (-1.03 - 1.18)
Elementary school	3.26 (2.19 - 4.32)
Middle school	5.05 (3.91 - 6.18)
High school	7.53 (5.99 - 9.08)
Vocational school	10.49 (8.12 - 12.86)
College and above	10.38 (7.86 - 12.89)
**Self-rated health**	
Excellent	0(Ref)
Very good	-2.8 (-4.46 - -1.15)
Good	-9.32 (-10.85 - -7.79)
Fair	-21.96 (-23.63 - -20.29)
Poor	-34.26 (-36.53 - -31.98)
**Number of NCDs**	
0	0(Ref)
1	-2.36 (-3.26 - -1.45)
>=2	-5.76 (-6.67 - -4.84)

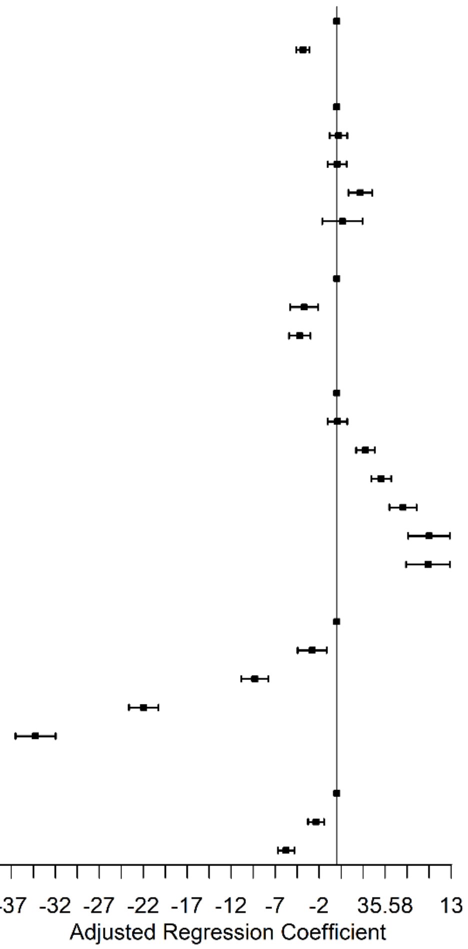

Figure S4e. Multiple linear regression between the psychology scale and sociodemographic and health factors.
